# Deep immune-phenotyping of HLA-homozygous iPS-cardiomyocytes by spectral flow cytometry

**DOI:** 10.3389/fimmu.2026.1736994

**Published:** 2026-02-04

**Authors:** Nicole Maeding, Deepika Suresh Kundully, Anna Steinhuber, Nils Kriedemann, Carlos A. Hernandez-Bautista, Soraia Martins, Sarah Hochmann, Martin Wolf, Wolfgang Mayr, Christof Jungbauer, Sebastian Diecke, Torsten Tonn, Boris Greber, Robert Zweigerdt, Dirk Strunk

**Affiliations:** 1 Austrian Red Cross Research, ÖRK, Vienna, Austria; 2 Cell Therapy Institute, Paracelsus Medical University, Salzburg, Austria; 3 Department of Cardiac, Thoracic, Transplantation and Vascular Surgery (HTTG), Leibniz Research Laboratories for Biotechnology and Artificial Organs, Research Center for Translational Regenerative Medicine, Hannover Medical School, Hannover, Germany; 4 Catalent Düsseldorf GmbH, Düsseldorf, Germany; 5 Austrian Red Cross, Blood Service for Vienna, Lower Austria and Burgenland, Vienna, Austria; 6 Department of Transfusion Medicine, University Hospital, Paracelsus Medical University, Salzburg, Austria; 7 Max-Delbrueck-Center for Molecular Medicine (MDC), Berlin, Germany; 8 Institute for Transfusion Medicine and Immunohematology, Goethe University Hospital Medical School, Frankfurt, Germany; 9 German Red Cross Blood Donor Service Baden-Wuerttemberg-Hessen, Frankfurt, Germany

**Keywords:** cardiomyocyte, hiPSC, immunophenotyping, regenerative medicine, spectral flow cytometry, transplantation immunology

## Abstract

**Introduction:**

Immunogenicity of allogeneic human induced pluripotent stem cell (hiPSC)-derived transplants limits their applicability in regenerative medicine. Selecting human leukocyte antigen (HLA)-homozygous hiPSC lines could be a mitigation strategy and haplo-matching would profoundly expand the number of potential recipients. Here we show deep immune-phenotyping of hiPSC-derived cardiomyocytes (iPS-CM) differentiated from four independent iPSC lines in three centers under chemically defined conditions.

**Methods and results:**

Broad immunophenotyping with 354 antibodies revealed differential expression of 101 immune-related molecules between iPS-CM and the parental hiPSC lines. We selected 54 key immune markers for deep immune-phenotyping by spectral flow cytometry at the single-cell level. We found that HLA-homozygous iPSCMs exhibit an overall stable immune-phenotype across HLA-homozygous and heterozygous hiPSC lines indicating a robust differentiation process. HLA-homozygous iPS-CM displayed significantly reduced HLA-ABC levels compared to heterozygous counterparts with an otherwise conserved immune-phenotype. Upon interferon gamma challenge as a surrogate of immune stress responsiveness, iPS-CM significantly upregulated HLA-ABC, -E, -F, PD-L1, PD-L2 and the 'don't eat me' signal CD47. As a proof-of-concept we used this panel to benchmark iPS-CM differentiation across three production sites in this study.

**Discussion:**

The data indicate generally stable immune-phenotype of iPS-CM produced at three different sites and support feasibility of monitoring iPS-CM identity by spectral flow cytometry.

## 1 Introduction

Cardiovascular diseases, including myocardial infarction and chronic heart failure remain the leading cause of morbidity and mortality worldwide. In the event of myocardial infarction an estimated one billion cardiomyocytes are terminally lost (1) and replaced by non-contractile, fibrotic tissue. The hiPSC technology (2) launched the promising approach of cell replacement therapy to substitute damaged or lost tissue (3–6). The capacity of iPS-CM to remuscularize damaged heart tissue has been shown in several pre-clinical studies using myocardial infarction animal models (7–11) and in clinical research using engineered heart muscle allografts in humans (7). Since hiPSCs can be derived in a patient-specific manner they have the potential to be used for autologous transplantation. The cost- and time-intensive generation of patient-specific hiPSCs and complex regulatory requirements hinder implementation. Allogeneic transplants are under intense investigation as an alternative since these would provide several advantages such as off-the-shelf availability, scalability of the production process, and cost effectiveness. From an immunological perspective the allogeneic approach is challenging due to HLA-dependent allograft recognition and subsequent rejection by the recipients’ immune system (12). Although direct allograft recognition of hiPSC-derived grafts may be avoided by the absence of passenger leukocytes, indirect and semi-direct allograft recognition as well as innate and NK-mediated immunity may induce graft rejection (13–15). As with conventional transplantation this can be mitigated by HLA matching (16), however, the high polymorphism of HLA genes leads to limited matching frequencies thus reducing the probability to find a suitable donor.

Allograft recognition by a recipients’ immune system and the ensuing rejection of the graft make allogeneic transplantation approaches challenging. To overcome this issue and reduce immunogenicity of hiPSCs-derivatives, several strategies have been employed, mostly based on genetic engineering (17). A common approach is deletion of beta-2 microglobulin which constitutes the light chain of the HLA class I heterodimer, and knockout of the class II transactivator for disrupting HLA class II (18–24). The complete lack of HLA molecules may, however, trigger activation of NK cells which respond to “missing self” (25). It also abrogates the ability of antigen presentation and subsequent target elimination in case of infection or oncogenic transformation (26–28). Consequently, several groups investigated editing of individual HLA genes to generate hypo-immunogenic hiPSCs pseudo-homozygous for HLA class I genes or deficient for HLA-A and -B (29–33). Other strategies increased immune compatibility by additional overexpression of inhibitory ligands, namely HLA-E, HLA-G, PD-L1 and 2, or the “don’t eat me” signal CD47 (30, 34–39).

In recent years there has been momentum to set up hiPSC banks derived from HLA-homozygous donors (40). The use of HLA-homozygous cells dramatically increases the donor/recipient matching frequency since only one of the recipients’ haplotypes needs to be matched (41, 42). This strategy can reduce costs and time requirements compared to autologous approaches enabling off-the-shelf availability of clinical grade hiPSCs and desired differentiated progeny cell therapy products. It leaves the inherent immunological properties intact thereby avoiding developmental costs and additional risks such as off-target effects associated with engineered cells. HLA-homozygous hiPSC-derived retinal pigment epithelial cells have been successfully transplanted in allogeneic settings without additional immunosuppression (43). However, studies in mice suggest organ-specific differences in the immune response to the graft. Bone marrow, neuronal cell, endothelial cells, dermal cells as well as hepatocytes were interestingly not rejected in syngeneic recipients. In contrast, cardiomyocytes induced a T cell response against the graft (44–47). Single-cell immune-phenotype data on PSC-derived cardiomyocytes are scarce with just one study on embryonic stem-cell-derived cardiomyocytes (48) and a comprehensive bulk transcriptomic and proteomic profiling of iPS-CM subtypes including selected flow cytometry data (49).

In this study, we therefore investigated the largely unexplored surface molecule composition of iPS-CMs at the single-cell level with a particular focus on molecules relevant to the immune response during host-graft interaction. Global immune-landscape screening upon iPS-CM differentiation revealed downregulation of HLA-ABC and beta-2 microglobulin (β2m), pluripotency markers Tra-1-60, Tra-2-49/-54, SSEA-5, and primed state hiPSC markers CD24 and CD90. We found downregulation of B7-H3 (CD276), NK ligand MICA/B, and of inducible T-cell co-stimulator ligand B7-H2 (CD275), and upregulation of MUC24 (CD164), N-cadherin (CD325), among others. We devised a 60-marker spectral flow cytometry panel in three test tubes that covers 54 immunophenotype and pluripotency markers to monitor the selected immune response profile upon differentiation of hiPSC into iPS-CM together with testing for lack of hiPSC marker-expressing cells in the final cell product, respectively. Each of the three test tubes contained anti-cTNT and viability stain as a reference in addition. Spectral typing confirmed uniform expression of CD164, B7-H3, CD112, and CD155, and significantly lower HLA class I expression in HLA-homozygous compared to heterozygous iPS-CM. Upon IFN-γ challenge we observed significant upregulation of HLA-ABC, HLA-DR, HLA-F and HLA-E, CD47, and PD-L1 (CD274) and PD-L2 (CD273) in iPS-CM. As a proof-of-concept we used this panel to benchmark iPS-CM differentiation across three production sites in this study as part of the european research consortion www.heal-horizon.com.

## 2 Materials and methods

### 2.1 HLA-homozygous and -heterozygous hiPSC

We used the following hiPSC lines: PMUi001-B (PMU1; PMUi001-A · Cell Line · hPSCreg) (50), R26, R26 HLA class I and class II double-knockout (R26^DKO^) (51), MDCi246-C, and MDCi055-C. All lines were generated previously using integration-free Sendai virus reprogramming. R26 is homozygous for 6/6 HLA alleles (HLA-A, -B, -C, -DRB1, -DQB1, -DPB1) at 6-digit resolution. R26^DKO^ is a genetically engineered sub-clone of R26 lacking major histocompatibility complex (MHC) class I (HLA-A, -B, -C) and class II (-HLA-DR, -DQ, - DP). MDCi246 is 6/6 homozygous, and MDCi055-C is 5/5 homozygous with HLA-DP mismatch. PMU1 was included as a heterozygous control (**Supplementary Table S1**). Detailed information on PMU and MDC hiPSC lines have been deposited at https://hpscreg.eu/.

### 2.2 HLA typing

HLA-typing of hiPSC was performed by next generation sequencing (NGS). Genomic DNA was extracted from cell culture samples using the Maxwell 16 Blood DNA Purification Kit (Promega). Library preparation of the six loci HLA-A, -B, -C, -DRB1, -DQB1, -DPB1 was performed using the HLA NGSgo workflow kit (GenDx). Sequencing of the libraries was performed using MiSeq v2 chemistry (2 x 150 cycles) on a MiSeq sequencer (Illumina). Sequencing data were analyzed in the NGSengine software (version 2.29.0.28288, GenDx) and library (version IMGT 3.51.0).

### 2.3 Culture of hiPSC

Human iPSCs were cultured at 37°C, 5% O_2_, 5% CO_2_ and saturated humidity in Essential 8 (E8) medium composed of DMEM/F12 (Gibco), 15 mM HEPES (Sigma), 100 ng/mL FGF2-G3 (Qkine), 2 ng/mL TGFβ-1 (Peprotech), 10.7 µg/mL transferrin, 64 µg/mL L-ascorbic acid-2-phophate, 14 ng/mL NaSeO3, and 20 µg/mL insulin; all Sigma). To eliminate the need for daily medium change we replaced FGF2 with thermostable FGF2-G3 (52, 53). Cells were passaged twice weekly at approximately 75% confluency. For passaging, cells were detached with Accutase (Sigma), washed with unsupplemented DMEM/F12, and reseeded at 1.2 x 10^4^ cells/cm^2^ for a 72 h culture period and 0.6 x 10^4^ cells/cm^2^ for a 96 h period. Optimal seeding density may vary depending on the specific iPSC line used and needs to be tested accordingly. Cells were reseeded into Matrigel^®^-coated flasks in E8 medium supplemented with 10 µM Y-27632 Rho kinase inhibitor (ROCKi; MedChemExpress). Medium was changed to E8 without ROCKi after 24 h. For IFN-γ treatment, differentiated iPS-CM aggregates containing 1 x 10^6^ cells were transferred to one well of a 12-well suspension plate. Culture medium was carefully aspirated and aggregates were washed with 1.5 mL PBS before adding 1.5 mL CDM3 containing 10 ng/mL IFN-γ (Peprotech) for 72 h under continous agitation (100 rpm).

### 2.4 Generation of hiPSC-derived cardiomyocytes

**Figure 1:**
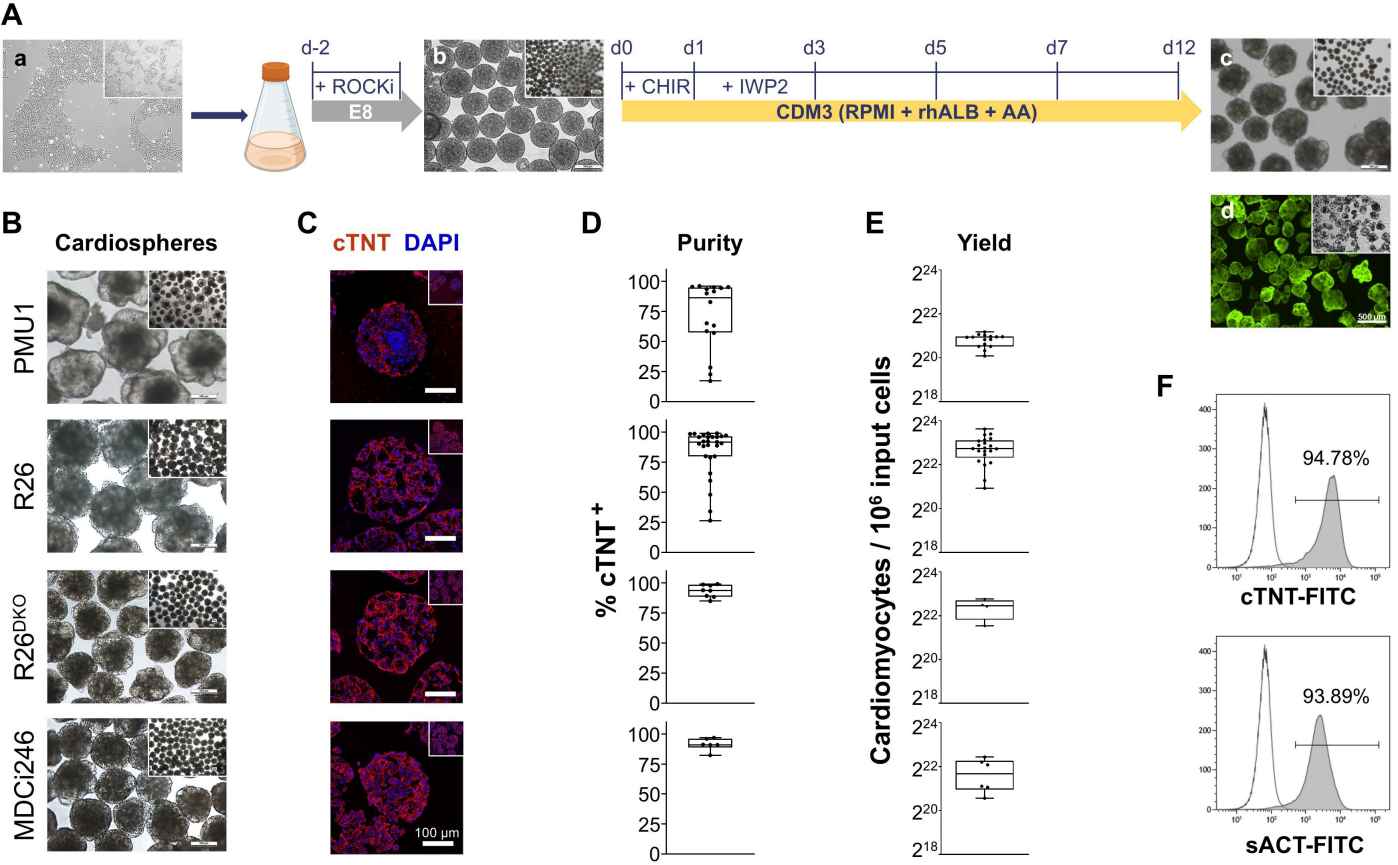
Differentiation of hiPSC into iPS-CM. **(A)** Experimental outline; representative pictures. **(a)** The hiPSC were released from 2D and aggregated in 3D culture in the presence of ROCKi. **(b)** Human iPSC aggregates were agitated in Erlenmeyer flasks at 70 rpm; cardiac differentiation was induced by 24-h WNT activation by CHIR99021 followed by 48-h WNT inhibition by IWP2, producing **(c)** iPS-CM aggregates (cardiospheres) homogenously positive for **(d)** calcein green fluorescence, indicating high viability. **(B)** Representative bright-field pictures of iPS-CM aggregates of four independent hiPSC lines at d12 as indicated with low resolution overview inserts. **(C)** Corresponding confocal microscopy images of fixed aggregates from **(B)** stained for cardiac troponin T (cTNT) (red) and nuclei (DAPI, blue; all scale bars 100 µm). **(D)** Purity of iPS-CM measured by flow cytometry as % cTNT; n = 6 – 25. **(E)** Yield of iPS-CM per million input iPSCs on d12 of differentiation; n = 4 - 21 **(F)** Representative flow cytometry histograms indicating purity of iPS-CM as measured by cytoplasmic cTNT and sarcomeric α-actinin (sACT) reactivity.

Differentiation of iPSC-derived cardiomyocytes in 3D suspension culture was carried out as previously described (54, 55). Briefly, hiPSCs were harvested from 60 – 70% confluent 2D cultures on day 2 using Accutase and transferred into 125 mL Erlenmeyer flasks (Corning; working volume 20 mL/flask) in E8 supplemented with 10 µM ROCKi. Inoculation density was 0.165 x 10^6^ cells/mL for R26, R26^DKO^, MDCi246, and MDCi55, and 0.33 x 10^6^ cells/mL for PMU1. Cultures were incubated for aggregate formation under continuous agitation (70 rpm). At day 0 medium was changed to CDM3 (RPMI1640, 15 mM HEPES, 2 mM L-glutamine, 495 µg/mL recombinant human serum albumin (Provitro), and 213 µg/mL L-ascorbic acid-2-phosphate) (56) containing 5 µM ROCKi. Cardiac differentiation was initiated through WNT activation by 5 µM CHIR99021; after 24 hours medium was exchanged for CDM3 plus 5 mM IWP-2 (both MedChemExpress) for WNT inhibition. 48 hours later medium was replaced by pure CDM3 and changed every other day as described previously in detail (54) (**Figure 1A**).

### 2.5 Control cells

Human peripheral blood mononuclear cells (PBMC) were obtained from healthy volunteers as described (50) to be used as a reference control cell mixture for spectral flow cytometry expressing various hematopoietic cell molecules. The Jurkat cell line was used as another reference representing a T cell type of mesodermal origin in deep immune-phenotyping. Jurkat cell stocks were purchased from the German Collection of Microorganisms and Cell Cultures (DSMZ; Jurkat ACC282) and expanded in RPMI-1640 (Sigma-Aldrich) containing 10% FBS (Gibco), 5 mM N(2)-L-Alanyl-L-Glutamin (Dipeptiven, Fresenius Kabi), and 10 mM HEPES (Sigma-Aldrich) before cryopreservation in a working cell bank at -170°C until further use in experiments.

### 2.6 Immunofluorescence staining and confocal microscopy

Deparaffinized iPS-CM aggregate FFPE sections were used for immunofluorescence staining. Antigen retrieval was performed using a 10 mM citrate buffer at pH = 6 (citric acid monohydrate, C1909, Sigma Aldrich) at 60°C for 3 hours, washing PBS and blocking for one hour in PBS/10% FBS (Gibco). The primary antibody (anti-troponin T, clone 13-11MA5-12960; Invitrogen) was incubated over night at 4°C in blocking buffer. Secondary antibody (goat anti-mouse IgG, Alexa Fluor 555, Thermo Fisher Scientific) and DAPI were incubated for one hour at room temperature in blocking buffer. Slides were mounted using the ProLong Gold anitfade reagent (P36934, Invitrogen). Confocal microscopy was performed using an Axio Observer Z1 laser-scanning microscope attached to LSM700 (Carl Zeiss).

### 2.7 Cell surface marker screening

A test kit covering 354 surface molecules was used according to the manufacturer’s instructions (LegendScreen™ Human PE kit, Biolegend). Briefly, cells were stained and fixed prior to acquisition. Samples were acquired on a Beckman Coulter Gallios™ flow cytometer. Data analysis was done using Kaluza 2.1 software. Expression levels were calculated relative to the corresponding isotype controls and displayed using the R package ComplexHeatmap.

### 2.8 Spectral flow cytometry

For deep immune-phenotyping, 1 x 10^6^ cells in 100 µL PBS were incubated with an antibody cocktail against selected surface molecules for 30 minutes at 4°C in the dark (**Supplementary Table S2**). After washing, cells were fixed in permeabilization buffer (eBioscience™ Thermo Fisher Scientific) for 30 minutes. For intracellular cardiac troponin T staining (cTNT-BV421, clone 13-11; Becton Dickinson), samples were incubated for 30 minutes with the antibody in permeabilization buffer. Samples were then washed, resuspended in PBS and acquired on a Cytek Northern Lights™ spectral flow cytometer equipped with 3 lasers (405, 488, and 640 nm). Data analysis was carried out using FCS Express 7 software (*De Novo* Software).

## 3 Results

### 3.1 Human iPSC line characterization and differentiation into iPS-CM

First, R26 was confirmed to be homozygous for 6/6 HLA alleles (HLA-A, -B, -C, -DRB1, -DQB1, -DPB1) at 6-digit resolution. MDCi246 was confirmed 6/6 homozygous, and MDCi055-C as 5/5 homozygous with HLA-DP mismatch. PMU1 was confirmed to be heterozygous for all six HLA loci and was included as a heterozygous control (**Supplementary Table S1**). R26 and MDCi246 represent the 2^nd^ and 24^th^ most frequent haplotypes in the German population with frequencies of 1.9949% and 0.2259%, respectively (http://www.allelefrequencies.net).

All lines showed typical hiPSC morphology with growth in colonies, sharply defined colony borders and high nucleus-to-cytoplasm ratios when cultured in 2D. Cardiac differentiation in suspension culture produced iPS-CM aggregates homogenously positive for calcein indicating high viability (**Figure 1A**). By day 12, iPS-CM aggregates from all lines increased in size compared to hiPSC aggregates and displayed typical cystic morphology (**Figure 1B**). Fixed aggregates stained positive for cTNT with median purities ranging from 86.45% ± 28.12% to 93.82% ± 5.24% (**Figures 1C, D**). In terms of differentiation efficiency, R26, R26^DKO^, MDCi246 and PMU1 yielded 6.96 ± 2.73 x 10^6^, 5.41 ± 1.71 x 10^6^, 3.49 ± 1.70 x 10^6^ and 2.00 ± 0.35 x 10^6^ iPS-CMs per million input cells, respectively (**Figure 1E**). Staining for cardiac markers cTNT and sarcomeric α-actinin (sACT) confirmed cardiac identity and purity of differentiated cells (**Figure 1F**).

### 3.2 Immune landscape screening

**Figure 2:**
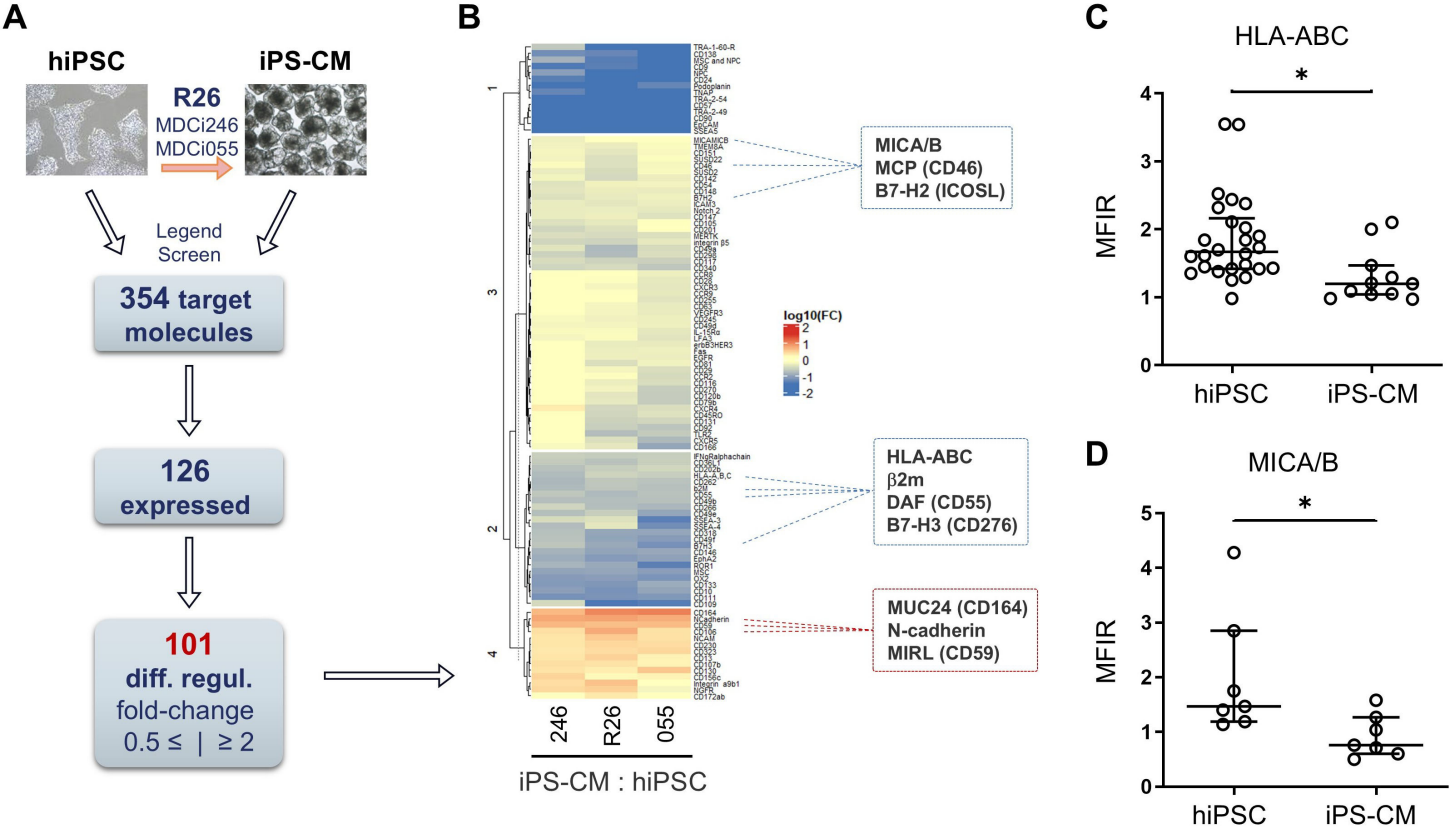
Broad immune-phenotype screening of hiPSC and iPS-CM. **(A)** Schematic outline of the screening array comparing HLA-homozygous hiPSC with their iPS-CM progeny as indicated (MDCi246, R26, MDCi055). **(B)** Heatmap representing the 101 significantly differentially regulated molecules (iPS-CM/hiPSC). Selected immune-recognition, -activation or -suppression molecules highlighted in boxes (red color/boxes: upregulated in iPS-CM, blue: downregulated in iPS-CM). **(C)** HLA-ABC and **(D)** MICA/B mean fluorescence intensity ratio (MFIR) calculated by dividing specific antibody staining and respective isotype control (n = 7 - 26; *p < 0.05, unpaired Student’s t-test, two-tailed).

We used a flow cytometry screening array covering 271 of the 371 currently classified CD molecules and 83 additional immune-relevant surface markers including HLA-ABC, HLA-DR, HLA-E, HLA-F and the natural killer (NK) cell ligands MHC class I chain-related protein A and B (MICA/B) to display the surface immune molecule landscape of d12 iPS-CM compared to parental hiPSC. Positive expression was defined as mean fluorescence intensity ratio (MFIR, MFI target/MFI isotype control) ≥ 2. Fold-changes between iPS-CM and hiPSC (MFIR iPS-CM/MFIR hiPSC) were used to identify differentially expressed markers defined as fold-change ≤ 0.5 and ≥ 2.0. To verify that the results are representative for HLA-homozygous iPS-CM, we included MDCi246 and MDCi055 in selected assays in addition to R26. Out of the 354 targets, 126 molecules were expressed either on hiPSC or corresponding iPS-CMs. Differential expression was found for 101 molecules (**Figure 2A**). K-means clustering of this dataset yielded four distinct clusters with three clusters (1 – 3) consisting of markers downregulated in iPS-CM while one cluster (4) contained upregulated markers. In line with their differentiation, iPS-CM downregulated molecules typically associated with undifferentiated cells and/or pluripotency such as Tra-1-60-R, SSEA-5, and Tra-2-49/-54 (cluster 1, median fold-change 0.016 ± 0.039). Likewise, CD24 and CD90 associated with a primed state of hiPSC were downregulated. Cluster 2 contained moderately downregulated surface markers (median fold-change 0.150 ± 0.085). The pluripotency marker SSEA-3 as well other markers associated with stem-like properties such as CD49f and CD133 were located in this cluster. In addition, several immunologically relevant markers were downregulated, including HLA-ABC and β2m which constitute the components of the major histocompatibility complex I (MHC I), as well as B7-H3 (CD276) and decay accelerating factor (DAF, CD55), both involved in the modulation of adaptive and innate immune responses.

Slightly downregulated molecules accounted for cluster 3 (median fold-change 0.481 ± 0.226). These included the NK ligand MICA/B, inducible T-cell co-stimulator ligand (ICOSL, B7-H2), and the complement-regulatory membrane cofactor protein CD46 (MCP). Cluster 4 included molecules upregulated in iPS-CM (median fold-change 2.438 ± 2.127). Among these were the cell adhesion molecule MUC24 (CD164), N-cadherin (CD325) as a central component of adherens junctions between cardiomyocytes, and the complement-inhibitory membrane inhibitor of reactive lysis (MIRL, CD59) (**Figures 2A, B**). To verify downregulation of HLA-ABC and MICA/B, two molecules integral to the interaction with T cells and NK cells, respectively, we performed additional single-staining. Both showed low levels of expression in hiPSC with a median MFIR 1.67 ± 0.63 and 1.47 ± 1.15. Upon differentiation into cardiomyocytes both molecules were significantly downregulated to MFIR of 1.20 ± 0.39 and 0.76 ± 0.39 close to the background level of the isotype control (**Figures 2C, D**).

### 3.3 Deep immune-phenotyping of iPS-CM by spectral flow cytometry

Based on the broad immunophenotyping results we devised a 60-marker deep immune-phenotyping panel comprising 54 immune and pluripotency markers together with cTNT and viability stain in three tubes (**Supplementary Table S2**, panel p1-3). Markers were chosen based on screening array results (n = 24 for differential expression; n = 20 for reactivity with both hiPSC and iPS-CM). We opted to add supplemental markers with a putative role in cell transplantation immunology (n = 10) including molecules of relevance in antigen presentation (HLA-DR) and during cellular stress and inflammation (e.g. HLA-E and CD274). The first tube was used for quality control (QC) containing markers to identify iPS-CM, hiPSC, hematopoietic cells, and endothelial cells. Testing on Jurkat cells, hiPSC, and iPS-CM showed clear separation of the different cell types in t-stochastic neighbourhood embedding (tSNE) analysis. We observed that the cTNT-negative population in the third iPS-CM batch formed an independent cluster (**Supplementary Figure S1A-C**). Cells in the individual clusters had distinct marker expression profile corresponding to the different cell types. Comparison of the cTNT-negative population in a randomly selected sample (9.18%) showed no significant overlap with the marker profiles of hiPSC or Jurkat cells (**Supplementary Figure S1D**). We tested iPS-CM differentiation batches for purity and only differentiations with a purity ≥ 80% cTNT reactivity were used for further experiments.

**Figure 3:**
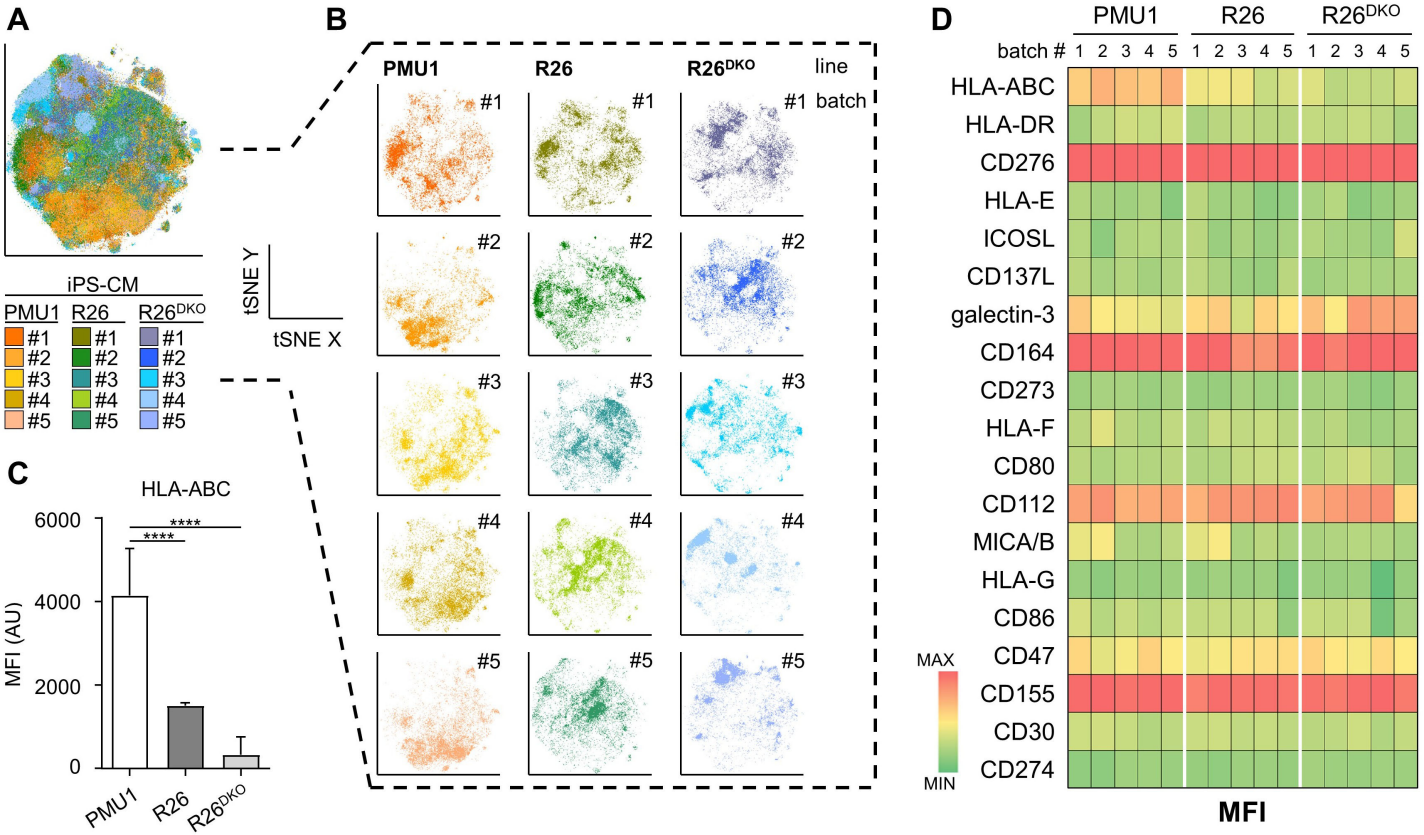
Deep immune-phenotyping of hiPSC and iPS-CM. **(A)** Dimensionality reduction (tSNE) of d12 iPS-CM from five independent differentiations per strain (PMU1, R26, R26^DKO^) based on 21-parameter expression profile (18 markers shown + cTNT and viability). **(B)** Depiction of the individual samples from **(A)**. **(C)** HLA-ABC expression summary as median fluorescence intensity (MFI) ± SD (n = 5; unpaired Student’s t-test, two-tailed, ****p ≤ 0.0001). **(D)** Heatmap representing the overall marker expression profile of each individual sample. Analyses included only cTNT^+^ cells.

To determine the immune-phenotype we used a 21-marker ‘immune recognition’ tube (tube 2; 19 immune markers plus cTNT and viability stain) and analyzed iPS-CM from five independent differentiation batches per hiPSC line. Using tSNE on the concatenated dataset we found that cells from individual batches were highly interspersed with no hiPSC line-specific clustering (**Figures 3A, B**). In HLA-heterozygous control iPS-CM (PMU1), HLA-ABC showed uniform moderate expression. The iPS-CM from HLA-homozygous R26 showed significantly lower HLA-ABC expression with expression levels slightly above background levels in R26^DKO^ (**Figure 3C**). Expression patterns of all other markers displayed no significant differences between the iPS-CM from different hiPSC lines and independent differentiation batches (**Figure 3D**). The iPS-CM from all three lines uniformly expressed B7-H3 (CD276; MFI = 39,565 ± 9,361), CD164 (MFI = 16,437 ± 5,282), CD112 (MFI = 4,973 ± 1,338), and CD155 (MFI = 12,288 ± 1,669). We found variable expression of galectin-3 (MFI range 739.49 to 6,444.83) and CD47 (MFI range 972.56 to 2,350.61) across independent differentiation batches but no hiPSC line-dependent differences. MICA/B was expressed at low level in two differentiation batches, one from PMU1 and R26 (MFI = 1,046 ± 164.5) but was negative in all other differentiations (MFI = 252.8 ± 73.24).

Testing on four independent differentiation batches of R26 (iPS-CM 1-4) using Jurkat cells as a non-myocyte reference reproducibly distinguished cTNT^+^ cardiomyocytes from cTNT-negative cells (**Supplementary Figure S2A**). Main driver of discrimination of cTNT-negatives was, besides cTNT, the absence of NCAM (CD56) and decreased expression VCAM-1 (CD106; MFI = 1,389.82 vs 2,473.34 to 10,325.10 in cTNT^+^ clusters) (**Supplementary Figure S2A**, cluster 3). We identified batch-dependent variation between batches regarding expression of SIRPα (CD172a) and VCAM-1: cTNT^+^ cardiomyocytes in cluster 1.1 and 1.2 (iPS-CM 1-3) showed lower expression of SIRPα compared to those in cluster 2.1 and 2.2, belonging to iPS-CM 4 (MFI = 3,596.79 and 3,066 vs 20,175.50 and 18,634.80). Similarly, expression of VCAM-1 was lower in iPS-CM from cluster 1.1 and 1.2 compared to those in cluster 2.1 and 2.2 (MFI = 4,048.59 and 4,156.21 vs 10,325.10 and 10,112.30). Furthermore, we identified a small iPS-CM subpopulation in all four batches (1.42% - 8.44%) with increased expression of CD235a (GYPA; MFI = 16,009 in cluster 1.2 and 24,686.30 in cluster 2.2), which was driving the separation from the main population. The main population of iPS-CM from all batches (cluster 1.1 and 2.1) were negative for CD235a (**Supplementary Figure S2B**). In addition, we detected a small subpopulation in batch #4 (9.64%), which was characterized by decreased expression of SIRPα and VCAM-1 and higher levels of CXCR4. All other markers tested were not expressed under baseline conditions.

### 3.4 Immune-phenotype alterations under pro-inflammatory conditions

**Figure 4:**
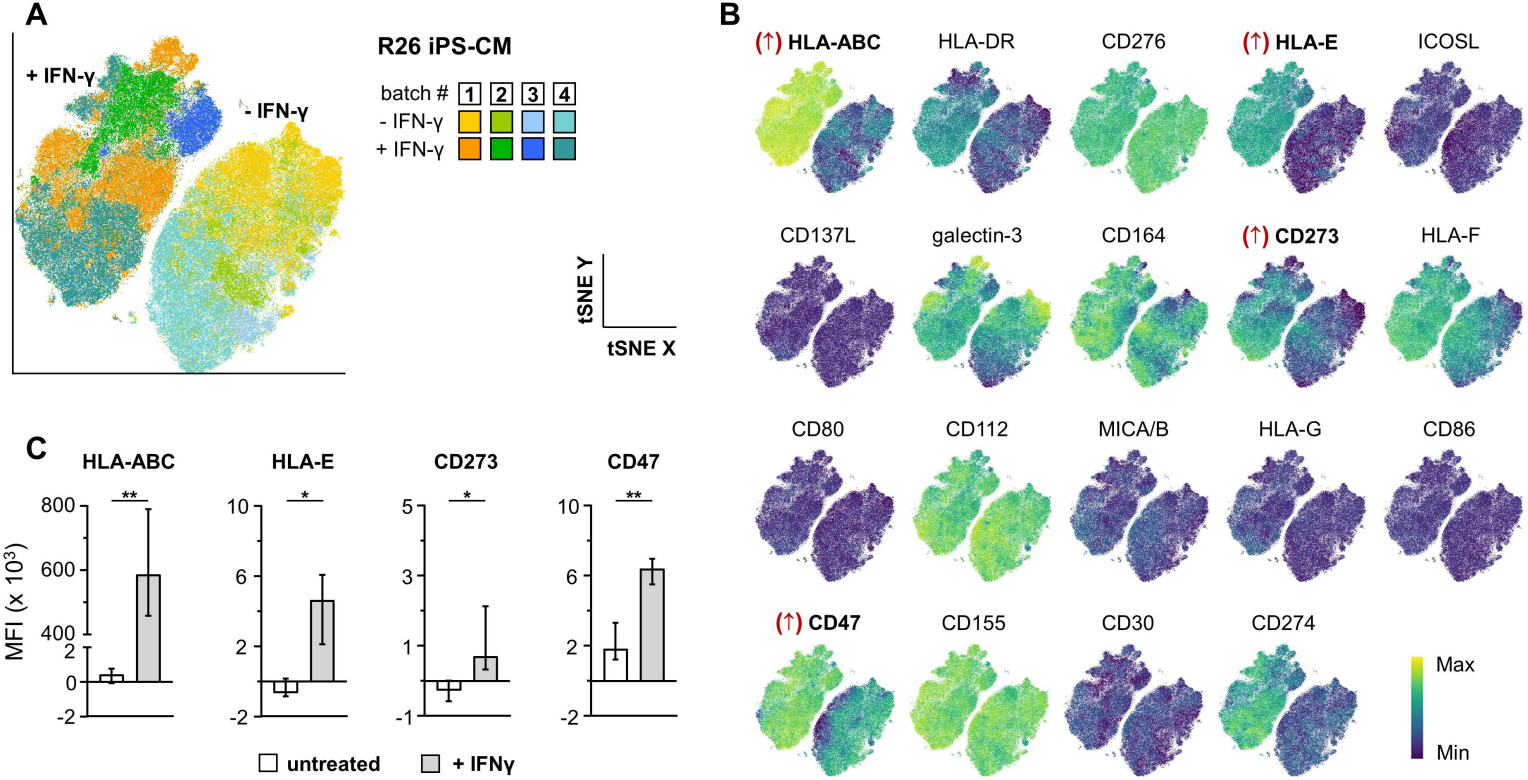
Response of iPS-CM to pro-inflammatory stimulation. **(A)** Clustering of HLA-homozygous R26 iPS-CM from four independent batches unstimulated or stimulated with IFN-γ. Color-coding represents sample identities. **(B)** Clustering from **(A)** resolved by expression heat of the individual markers. **(C)** Induction of HLA-ABC, HLA-E, PD-L2 (CD273), and CD47 (paired Student’s t-test, two-tailed; *p ≤ 0.05, **p ≤ 0.01). Analyses included only cTNT^+^ cells.

Upon transplantation iPS-CM may be exposed to a variety of microenvironmental cues including pro-inflammatory signals. We opted for an IFN-γ challenge of hiPS-CM as a prototypic stimulus to test the impact of pro-inflammatory signaling on their immune phenotype. IFN-γ-treated and -untreated iPS-CM clearly separated from each other (**Figure 4A**). We observed significant upregulation of HLA-ABC, HLA-E, CD47, and PD-L2 (CD273) in IFN-γ-treated samples (**Figures 4B, C**). HLA-DR, HLA-F and PD-L1 (CD274) showed increased expression that was not significant in this small dataset. The immune recognition panel also enabled identification of aberrant differentiation batches: two of the investigated unstimulated samples (batch #5 and #6) clustered separately in the tSNE (**Supplementary Figure S3A**). Individual marker expression analysis showed decreased levels of CD276 for both samples, which was maintained in the stimulated sample from batch #5). In addition, untreated samples from batch #5 had reduced levels of CD164, CD112, CD47, and CD155 (**Supplementary Figure S3B**). Upon IFN-γ treatment all identified markers showed expression levels comparable to those seen in samples from batch #1–4 indicating a regular response to pro-inflammatory signals (**Supplementary Figure S3B**). Of note, when the two samples from batch #5 and #6 were included in the overall statistical analysis, also HLA-DR, HLA-F and PD-L1 (CD274) were significantly upregulated upon IFN-γ treatment (**Supplementary Figures S3B, C**).

### 3.5 Stability of the immune-phenotype across different production sites

**Figure 5:**
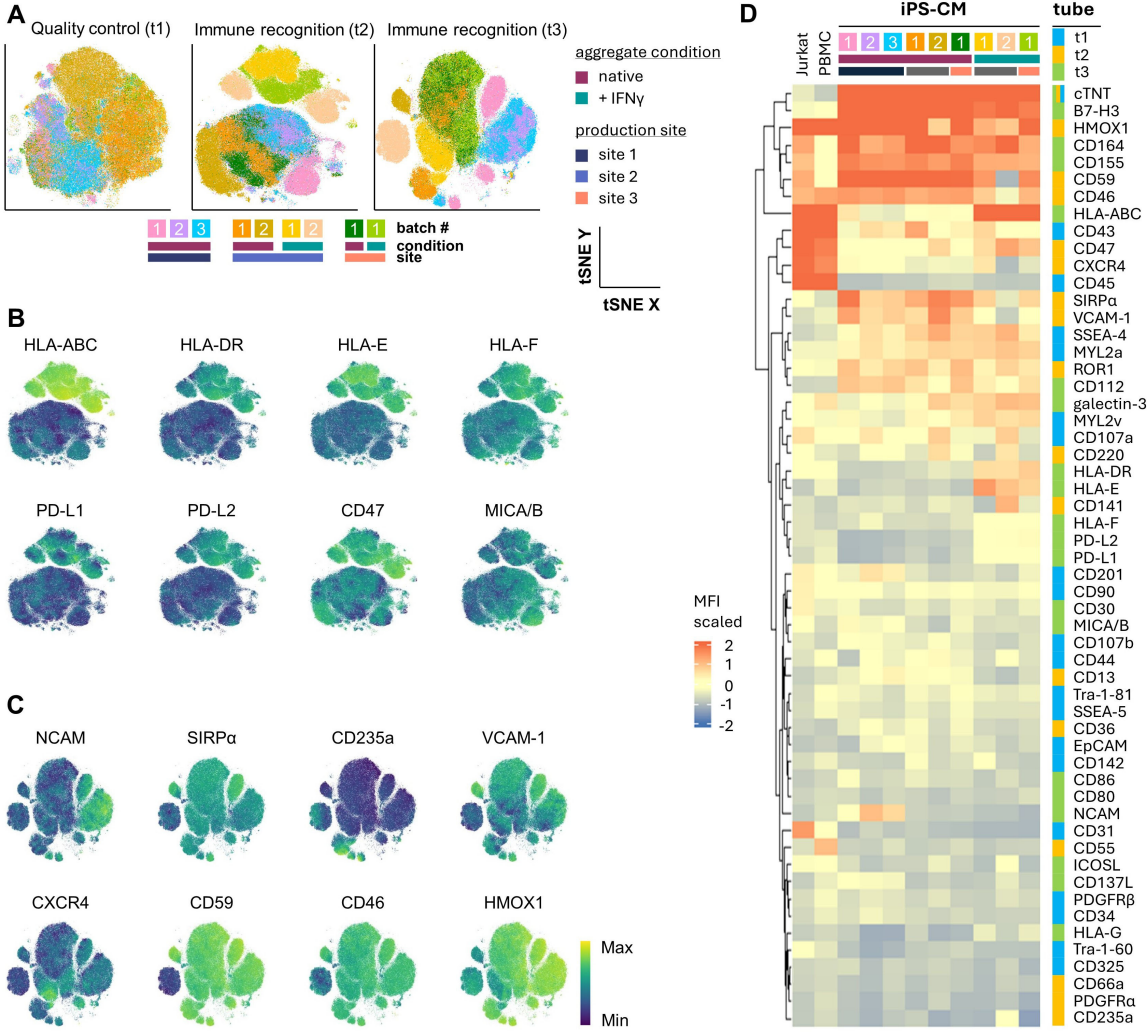
Stability of the iPS-CM immune-phenotype across different production sites. **(A)** Dimensionality reduction (tSNE) of 6 independent iPS-CM differentiations of HLA-homozygous R26 performed at 3 different production sites with and without final IFN-γ challenge. Separate depiction of marker combination in three spectral flow cytometry analysis tubes (t1-3) as shown. Color-coding represents sample identity. **(B)** Tube 2 and **(C)** tube 3 tSNE blots showing the expression heat of markers driving the separation of clusters in samples. **(D)** Heatmap showing the overall expression profiles of viable cells in samples from individual differentiation batches across all 54 markers plus cTNT. Marker profiles of Jurkat cells and PBMCs were included as a reference. Median fluorescence intensity (MFI) was scaled column-wise.

Phenotypic stability across different production sites is a requirement for efficient and reproducible clinical translation of cell therapy products. We used independent R26 iPS-CM differentiation batches from three different production sites to test the stability of the resulting iPS-CM immune-phenotype. Dimensionality reduction of the multidimensional spectral flow cytometry data from the different batches confirmed immunophenotypic homogeneity across the three different sites as it did not show any specific clustering corresponding to a respective production site. We confirmed separation of IFN-γ-treated from untreated samples (**Figure 5A**). This separation was again driven, as observed in previous experiments in this study, by significant upregulation of HLA-ABC (MFI = 478,053 treated vs 1,136 untreated; p < 0.0001), HLA-DR (MFI = 5,530 treated vs -156.8 untreated; p < 0.0001), HLA-E (MFI = 5,225 treated vs -143.7 untreated; p = 0.0023), HLA-F (MFI = 1,779 treated vs 385.9 untreated; p = 0.0003), PD-L1 (MFI = 1,137 treated vs -137.2 untreated; p < 0.0001), PD-L2 (MFI = 1,640 treated vs -117 untreated; p < 0.0001), and CD47 (MFI = 5,076 treated vs 1,342 untreated; p = 0.0076). MICA/B showed batch-dependent variation (MFI range 198 to 1509) but no treatment- or site-specific expression patterns (**Figure 5B**). Expression of NCAM (CD56), SIRPα (CD172a), VCAM-1 (CD106), CD59, CD46, and heme oxygenase 1 (HMOX1) varied between batches while CD235a and CXCR4 identified subpopulations within individual batches (**Figure 5C**).

High cTNT and B7-H3 expression, low HLA-ABC, CD43, CD47, CXCR4 and lack of CD45 expression discriminated unstimulated iPS-CM from Jurkat and PBMC. CD164, CD155 and CD59 showed higher expression in iPS-CM compared to Jurkat cells and were absent in PMBC. Notably, CD164 showed pronounced batch-dependent variability in iPS-CM. Similarly, iPS-CM expressed varying levels of SIRPα, VCAM-1, ROR1, and CD112 while Jurkat and PBMC did not. Galectin-3 and CD107a expression discriminated PC and Jurkat from each other and were variably expressed in iPS-CM. Immune-activation and -modulation-related markers showed a clear dependency on microenvironmental cues, i.e. stimulation with IFN-γ: HLA-ABC was strongly upregulated after IFN-γ treatment, reaching levels of Jurkat and PBMC. CD47, HLA-E, HLA-DR, HLA-F, PD-L1 and PD-L2 were upregulated as well. Importantly, co-stimulatory molecules CD80 and CD86 were not expressed. Data further illustrated lack of pluripotency markers corresponding to the differentiated state of the cells used. MYL2a/2v showed lower expression level in some but not all batches, compatible with an early differentiation state, irrespective of IFN-γ challenge. (**Figure 5D**).

## 4 Discussion

Here, we present an in-depth characterization of the immune-phenotype of HLA-homozygous and HLA-heterozygous iPS-CM under steady-state conditions and in response to IFN-γ. Our results demonstrate that iPS-CM generally display a surface marker profile with low expression of immune recognition molecules and intact responsiveness to proinflammatory cues. HLA-homozygous iPS-CM express significantly lower levels of HLA-ABC, which points towards reduced immunogenicity due to diminished allogeneic T-cell-mediated graft recognition. Overall, these data support the concept of HLA-homozygous iPS-CM as semi-universal donor cells in cardiac cell replacement therapy.

In line with cardiac specification, differentiated cells efficiently lost markers associated with pluripotency and stemness. Both components of MHC I, HLA-ABC and β2m, were significantly reduced in iPS-CM compared to parental hiPSC. Decreased HLA-ABC expression in hiPSC-derived cell products compared to parental iPSC has been reported in numerous studies (29, 57–63). In addition, HLA class I expression differs substantially between tissues and cell types and cardiomyocytes are known to express only very low levels of HLA class I under homeostatic conditions (64, 65). Didie et al. (66) found that even after further maturation of stem cell-derived cardiomyocytes MHC I levels did not increase. Thus, the observed downregulation of HLA-ABC appears to be intrinsic to cardiomyocytes rather than an artifact of *in vitro* differentiation protocols. Like HLA-ABC, additional immune-modulatory molecules such as DAF (CD55), MCP (CD46), and B7-H3 (CD276) were downregulated.

HLA-homozygous iPS-CM expressed significantly lower levels of MHC I compared to HLA-heterozygous counterparts. This may be attributed in part to differences in HLA expression based on the specific HLA allele utilised, for instance HLA-A*01:01:01 (R26) has been found to be expressed at lower levels than HLA-A*24:02:01 (PMU1) (67). Additional factors such as the production of alternative splice variants and gene dosage may contribute to the observed difference (68–70). Otherwise, we did not observe any specific clustering separating the three HLA-homozygous lines from the HLA-heterozygous PMU1. Immune-modulatory B7-H3, CD112, CD155, and CD47 were uniformly expressed across samples which highlights a stable baseline immune profile across genetic backgrounds. This immune profile together with the reduced expression of MHC I could be a favorable feature in a transplant setting due to potentially reduced allo-recognition. Together with the HLA-homozygous nature of the haplo-matched graft this could result in a decreased need for immunosuppression. However, studies in non-human primates indicate that a certain level of immunosuppression may still be necessary to avoid immune rejection (71). One underlying reason could be that reduced HLA class I levels may trigger activation of NK cells due to “missing-self” signaling. Reduced HLA levels may shift the balance in favor of cytotoxicity activating signals such as the stress-induced NK ligand MICA/B.

Microenvironmental cues affecting hiPSC-derived cell therapy products after application may include hypoxia and inflammation, among others. We tested proinflammatory stimulation with IFN-γ and observed a distinct immune activation response in iPS-CM. We observed significant upregulation of HLA-ABC, -E, -F, and -DR, CD47, and PD-L1 (CD274) and PD-L2 (CD273), indicating intact activation of interferon-responsive pathways to enhance antigen presentation and regulate immune checkpoint signalling. Although upregulation of most of these markers may be expected, these results demonstrate that HLA-homozygous iPS-CM remain reactive to proinflammatory cues and can adapt their immune-phenotype in response to environmental signalling. Of note, PD-L2 (CD273) expression in cardiomyocytes is not well documented but its inducibility by IFN-γ has been reported in other tissues. Our findings may indicate that iPS-CM can upregulate multiple immune-checkpoint ligands in response to proinflammatory signals. This may contribute to local immunomodulation to prevent damage to the graft via inhibition of PD-1-expressing infiltrating T cells. Moreover, to the best of our knowledge, our dataset provides the first high-dimensional, single-cell, 54-marker immune phenotype map of iPS-CM. Apart from analysis of classical HLA-ABC induction, our approach integrates non-classical HLA family members (HLA-E/F/G), co-stimulatory molecules, checkpoint ligands (PD-L1/2, CD47), and cardiomyocyte- and iPSC-specific markers within one unified single-cell framework. This comprehensive profiling, which was replicated across three production sites, demonstrates the robustness of the iPS-CM immune phenotype and provides a relevant reference for manufacturing and quality control.

We observed MICA/B expressed at low level in two differentiation batches, one each from PMU1 and R26, in our center. Also, in the manufacture site comparison, we observed batch-dependent MICA/B variation but no treatment- or site-specific expression patterns. MICA/B are polymorphic stress-induced NKG2D ligands which can be induced on cardiomyocytes upon ischemia-reperfusion injury and can be controlled by cyclosporin-dependent suppression of hypoxia-inducible transcription factors (72). We may speculate that MICA/B makes hiPSC and iPS-CM sensitive to NKG2D-mediated lysis. We therefore plan to focus on MICA/B monitoring during future functional studies. We also identified a < 10% iPS-CM subpopulation with increased expression of CD235a (GYPA) in several differentiation batches. GYPA was described to be transiently expressed early during ventricular specification (73) and may therefore document a heterogenous mixture of atrial and ventricular cardiomyocytes or be indicative of a slightly asynchronous differentiation from iPSC to iPS-CM within some of our batches. We also observed batch-dependent variability in the expression of VCAM-1 and SIRPα. Both molecules have been shown to be induced during iPS-CM differentiation (74) and their slightly lower levels in some batches may suggest a delay in iPS-CM development in those batches. Importantly, this variability did not segregate by production site, suggesting that it originates from subtle differentiation dynamics rather than center-specific workflows. Our spectral flow cytometry panel thus offers a sensitive method to detect these deviations and may support future establishment of release criteria defining acceptable phenotypic ranges for clinical-grade iPS-CM.

Heterogeneity of iPS-CM has been observed earlier particularly after single-cell RNA-seq enabling precise allocation of cardiac transcription factor profiles in individual cells or cell populations (75). These data have been interpreted as indicating earlier atrial compared to later ventricular Wnt-induced differentiation signature (75). Our data, for the first time, demonstrate limited phenotypic heterogeneity at the single-cell level with a high sensitivity based on typing 54-markers on each cTNT^+^ cell. Longer persistence of GYPA until d12 may be attributed to the conventional 3-day CHIR/IWP2 induction used in our center because optimal timed specification of atrial and ventricular has been shown to depend on more sophisticated mesoderm induction (73). Follow-up studies are therefore already underway comparing different more complex cardiogenic induction protocols. It may also be interesting to see future spectral flow cytometry-based iPS-CM typing by other centers and with improving protocols. Ideally, pure cell products are considered to be best suited for transplantation. We may speculate that sensitive single-cell multi-parameter could always detect minor degree of heterogeneity – an acceptable threshold needs to be determined in future studies. Also, the presumed phenotypic homogeneity of cardiomyocytes derived from a local biopsy needs to be confirmed with modern single-cell multi-parametric techniques, e.g. using the spectral panel published herein. Finally, the impact of limited impurity or if sort-purification (76) needs to be considered deserves prospective analysis.

This study has several limitations: Our marker selection, despite following rational panel design based on comprehensive legend screen for a broad immunophenotyping, just included 44 of the 101 significantly expressed immune markers. Additional antibody targets may be included in future panel design. Additional myocyte-specific targets may also be required because MYL2a and MYL2v in this study did not show strong signals in all iPS-CM; later time points of analysis beyond d12 may also be advantageous in future studies. A comprehensive analysis of functional immune-matching including cytotoxicity and CD4/CD8 activation and cytokine production is already in planning. Ideally, we will be able to discriminate indirect vs semi-direct putative immune responses in different matching scenarios using haplo-matched as compared to control mismatched responder lymphocytes. Another limitation relates to the fact that iPS-CM characteristically display an immature phenotype resembling rather fetal than adult cardiomyocytes. Future detailed functional studies are needed to determine the immunogenicity of iPS-CM *in vitro* and *in vivo*.

Taken together, the presented data demonstrate that iPS-CMs exhibit a generally stable immune-phenotype across HLA-homozygous and heterozygous lines with preserved responsiveness to IFN-γ. The current spectral flow cytometry panel was sensitive enough to detect small iPS-CM subpopulations based on expression of CD235a (GYPA), CD172a (SIRPα), CD56 (NCAM), CD13, and CD106. These results support the feasibility of monitoring the immune-phenotype of iPS-CMs by spectral flow cytometry in cardiac cell replacement therapy.

## Data Availability

The original contributions presented in the study are included in the article/**Supplementary Material**. Further inquiries can be directed to the corresponding authors.

## References

[1] Laflamme MA Murry CE . Heart regeneration. Nature. (2011) 473:326–35. doi: 10.1038/nature10147, PMID: 21593865 PMC4091722

[2] Takahashi K Yamanaka S . Induction of pluripotent stem cells from mouse embryonic and adult fibroblast cultures by defined factors. Cell. (2006) 126:663–76. doi: 10.1016/j.cell.2006.07.024, PMID: 16904174

[3] Mora C Serzanti M Consiglio A Memo M Dell’Era P . Clinical potentials of human pluripotent stem cells. Cell Biol Toxicol. (2017) 33:351–60. doi: 10.1007/s10565-017-9384-y, PMID: 28176010

[4] Burridge PW Keller G Gold JD Wu JC . Production of *de novo* cardiomyocytes: human pluripotent stem cell differentiation and direct reprogramming. Cell Stem Cell. (2012) 10:16–28. doi: 10.1016/j.stem.2011.12.013, PMID: 22226352 PMC3255078

[5] Takahashi K Tanabe K Ohnuki M Narita M Ichisaka T Tomoda K . Induction of pluripotent stem cells from adult human fibroblasts by defined factors. Cell. (2007) 131:861–72. doi: 10.1016/j.cell.2007.11.019, PMID: 18035408

[6] Laflamme MA Murry CE . Regenerating the heart. Nat Biotechnol. (2005) 23:845–56. doi: 10.1038/nbt1117, PMID: 16003373

[7] Jebran AF Seidler T Tiburcy M Daskalaki M Kutschka I Fujita B . Engineered heart muscle allografts for heart repair in primates and humans. Nature. (2025) 639:503–11. doi: 10.1038/s41586-024-08463-0, PMID: 39880949 PMC11903342

[8] Selvakumar D Reyes L Chong JJH . Cardiac cell therapy with pluripotent stem cell-derived cardiomyocytes: what has been done and what remains to do? Curr Cardiol Rep. (2022) 24:445–61. doi: 10.1007/s11886-022-01666-9, PMID: 35275365 PMC9068652

[9] Kawaguchi S Soma Y Nakajima K Kanazawa H Tohyama S Tabei R . Intramyocardial transplantation of human iPS cell–derived cardiac spheroids improves cardiac function in heart failure animals. JACC Basic Transl Sci. (2021) 6:239–54. doi: 10.1016/j.jacbts.2020.11.017, PMID: 33778211 PMC7987543

[10] Kashiyama N Miyagawa S Fukushima S Kawamura T Kawamura A Yoshida S . MHC-mismatched allotransplantation of induced pluripotent stem cell-derived cardiomyocyte sheets to improve cardiac function in a primate ischemic cardiomyopathy model. Transplantation. (2019) 103:1582–90. doi: 10.1097/TP.0000000000002765, PMID: 31107828

[11] Shiba Y Gomibuchi T Seto T Wada Y Ichimura H Tanaka Y . Allogeneic transplantation of iPS cell-derived cardiomyocytes regenerates primate hearts. Nature. (2016) 538:388–91. doi: 10.1038/nature19815, PMID: 27723741

[12] Charles A Janeway J Travers P Walport M Shlomchik MJ . Immunobiology. Immunobiology. (2001) 14102):1–10.

[13] Li Q Lan P . Activation of immune signals during organ transplantation. Signal Transduct Target Ther. (2023) 8:110. doi: 10.1038/s41392-023-01377-9, PMID: 36906586 PMC10008588

[14] Yatim N Cullen S Albert ML . Dying cells actively regulate adaptive immune responses. Nat Rev Immunol. (2017) 17:262–75. doi: 10.1038/nri.2017.9, PMID: 28287107

[15] Spörri R Reis e Sousa C . Inflammatory mediators are insufficient for full dendritic cell activation and promote expansion of CD4+ T cell populations lacking helper function. Nat Immunol. (2005) 6:163–70. doi: 10.1038/ni1162, PMID: 15654341

[16] Flomenberg N Baxter-Lowe LA Confer D Fernandez-Vina M Filipovich A Horowitz M . Impact of HLA class I and class II high-resolution matching on outcomes of unrelated donor bone marrow transplantation: HLA-C mismatching is associated with a strong adverse effect on transplantation outcome. Blood. (2004) 104:1923–30. doi: 10.1182/blood-2004-03-0803, PMID: 15191952

[17] Yanamandala M Zhu W Garry DJ Kamp TJ Hare JM Jun HW . Overcoming the roadblocks to cardiac cell therapy using tissue engineering. J Am Coll Cardiol. (2017) 70:766. doi: 10.1016/j.jacc.2017.06.012, PMID: 28774384 PMC5553556

[18] Wei Y Geng X You Q Zhang Y Cao F Narayanan G . Human induced pluripotent stem cell derived nanovesicles for cardiomyocyte protection and proliferation. Bioact Mater. (2025) 50:585–602. doi: 10.1016/j.bioactmat.2025.04.017, PMID: 40453695 PMC12124652

[19] Trionfini P Romano E Varinelli M Longaretti L Rizzo P Giampietro R . Hypoimmunogenic human pluripotent stem cells as a powerful tool for liver regenerative medicine. Int J Mol Sci. (2023) 24:11810. doi: 10.3390/ijms241411810, PMID: 37511568 PMC10380710

[20] Zha S Tay JCK Zhu S Li Z Du Z Wang S . Generation of mesenchymal stromal cells with low immunogenicity from human PBMC-derived β2 microglobulin knockout induced pluripotent stem cells. Cell Transplant. (2020) 29:963689720965529. doi: 10.1177/0963689720965529, PMID: 33172291 PMC7784598

[21] Norbnop P Ingrungruanglert P Israsena N Suphapeetiporn K Shotelersuk V . Generation and characterization of HLA-universal platelets derived from induced pluripotent stem cells. Sci Rep. (2020) 10:1–9. doi: 10.1038/s41598-020-65577-x, PMID: 32439978 PMC7242456

[22] Wang X Lu M Tian X Ren Y Li Y Xiang M . Diminished expression of major histocompatibility complex facilitates the use of human induced pluripotent stem cells in monkey. Stem Cell Res Ther. (2020) 11:1–14. doi: 10.1186/s13287-020-01847-9, PMID: 32746912 PMC7397609

[23] Mattapally S Pawlik KM Fast VG Zumaquero E Lund FE Randall TD . Human leukocyte antigen class I and II knockout human induced pluripotent stem cell–derived cells: Universal donor for cell therapy. J Am Heart Assoc. (2018) 7 (23):e010239. doi: 10.1161/JAHA.118.010239, PMID: 30488760 PMC6405542

[24] Feng Q Shabrani N Thon JN Huo H Thiel A Machlus KR . Scalable generation of universal platelets from human induced pluripotent stem cells. Stem Cell Rep. (2014) 3:817–31. doi: 10.1016/j.stemcr.2014.09.010, PMID: 25418726 PMC4235139

[25] Lanier LL . NK cell recognition. Annu Rev Immunol. (2005) 23:225–74. doi: 10.1146/annurev.immunol.23.021704.115526, PMID: 15771571

[26] Ichise H Nagano S Maeda T Miyazaki M Miyazaki Y Kojima H . NK Cell Alloreactivity against KIR-Ligand-Mismatched HLA-Haploidentical Tissue Derived from HLA Haplotype-Homozygous iPSCs. Stem Cell Rep. (2017) 9:853–67. doi: 10.1016/j.stemcr.2017.07.020, PMID: 28867344 PMC5599245

[27] McGranahan N Rosenthal R Hiley CT Rowan AJ Watkins TBK Wilson GA . Allele-specific HLA loss and immune escape in lung cancer evolution. Cell. (2017) 171:1259–1271.e11. doi: 10.1016/j.cell.2017.10.001, PMID: 29107330 PMC5720478

[28] Marty R Kaabinejadian S Rossell D Slifker MJ van de Haar J Engin HB . MHC-I genotype restricts the oncogenic mutational landscape. Cell. (2017) 171:1272–1283.e15. doi: 10.1016/j.cell.2017.09.050, PMID: 29107334 PMC5711564

[29] Kim J Nam Y Jeon D Choi Y Choi SJ Hong CP . Generation of hypoimmunogenic universal iPS cells through HLA-type gene knockout. Exp Mol Med. (2025) 57:686–99. doi: 10.1038/s12276-025-01422-3, PMID: 40087529 PMC11958689

[30] Han X Wang M Duan S Franco PJ Kenty JHR Hedrick P . Generation of hypoimmunogenic human pluripotent stem cells. Proc Natl Acad Sci U S A. (2019) 116:10441–6. doi: 10.1073/pnas.1902566116, PMID: 31040209 PMC6535035

[31] Xu H Wang B Ono M Kagita A Fujii K Sasakawa N . Targeted Disruption of HLA Genes via CRISPR-Cas9 Generates iPSCs with Enhanced Immune Compatibility. Cell Stem Cell. (2019) 24:566–578.e7. doi: 10.1016/j.stem.2019.02.005, PMID: 30853558

[32] Torikai H Reik A Soldner F Warren EH Yuen C Zhou Y . Toward eliminating HLA class I expression to generate universal cells from allogeneic donors. Blood. (2013) 122:1341–9. doi: 10.1182/blood-2013-03-478255, PMID: 23741009 PMC3750336

[33] Flahou C Morishima T Takizawa H Sugimoto N . Fit-for-all iPSC-derived cell therapies and their evaluation in humanized mice with NK cell immunity. Front Immunol. (2021) 12:662360. doi: 10.3389/fimmu.2021.662360, PMID: 33897711 PMC8059435

[34] Kim CY Jeong C Jeong YJ Sung YH Han Y Hwang C . Establishment of immune-evasive iPSCs from PBMCs using B2M knockout and CD47/HLA-E overexpression. Tissue Eng Regener Med. (2025) 22 (7):1005–17. doi: 10.1007/s13770-025-00742-9, PMID: 40682609 PMC12476349

[35] Tsuneyoshi N Hosoya T Takeno Y Saitoh K Murai H Amimoto N . Hypoimmunogenic human iPSCs expressing HLA-G, PD-L1, and PD-L2 evade innate and adaptive immunity. Stem Cell Res Ther. (2024) 15:1–20. doi: 10.1186/s13287-024-03810-4, PMID: 38956724 PMC11218117

[36] Wang B Iriguchi S Waseda M Ueda N Ueda T Xu H . Generation of hypoimmunogenic T cells from genetically engineered allogeneic human induced pluripotent stem cells. Nat Biomed Engineering. (2021) 5:429–40. doi: 10.1038/s41551-021-00730-z, PMID: 34002062

[37] Shi L Li W Liu Y Chen Z Hui Y Hao P . Generation of hypoimmunogenic human pluripotent stem cells via expression of membrane-bound and secreted β2m-HLA-G fusion proteins. Stem Cells. (2020) 38:1423–37. doi: 10.1002/stem.3269, PMID: 32930470

[38] Deuse T Hu X Gravina A Wang D Tediashvili G De C . Hypoimmunogenic derivatives of induced pluripotent stem cells evade immune rejection in fully immunocompetent allogeneic recipients. Nat Biotechnol. (2019) 37:252–8. doi: 10.1038/s41587-019-0016-3, PMID: 30778232 PMC6419516

[39] Gornalusse GG Hirata RK Funk SE Riolobos L Lopes VS Manske G . HLA-E-expressing pluripotent stem cells escape allogeneic responses and lysis by NK cells. Nat Biotechnol. (2017) 35:765–72. doi: 10.1038/nbt.3860, PMID: 28504668 PMC5548598

[40] Escribá R Beksac M Bennaceur-Griscelli A Glover JC Koskela S Latsoudis H . Current landscape of iPSC haplobanks. Stem Cell Rev Rep. (2024) 20:2155–64. doi: 10.1007/s12015-024-10783-7, PMID: 39276260 PMC11554736

[41] Taylor CJ Peacock S Chaudhry AN Bradley JA Bolton EM . Generating an iPSC bank for HLA-matched tissue transplantation based on known donor and recipient hla types. Cell Stem Cell. (2012) 11:147–52. doi: 10.1016/j.stem.2012.07.014, PMID: 22862941

[42] Nakatsuji N Nakajima F Tokunaga K . HLA-haplotype banking and iPS cells. Nat Biotechnol. (2008) 26:739–40. doi: 10.1038/nbt0708-739, PMID: 18612291

[43] Sugita S Mandai M Hirami Y Takagi S Maeda T Fujihara M . HLA-matched allogeneic iPS cells-derived RPE transplantation for macular degeneration. J Clin Med. (2020) 9:2217. doi: 10.3390/jcm9072217, PMID: 32668747 PMC7408794

[44] Zhao T Zhang ZN Westenskow PD Todorova D Hu Z Lin T . Humanized mice reveal differential immunogenicity of cells derived from autologous induced pluripotent stem cells. Cell Stem Cell. (2015) 17:353–9. doi: 10.1016/j.stem.2015.07.021, PMID: 26299572 PMC9721102

[45] De Almeida PE Meyer EH Kooreman NG Diecke S Dey D Sanchez-Freire V . Transplanted terminally differentiated induced pluripotent stem cells are accepted by immune mechanisms similar to self-tolerance. Nat Commun. (2014) 5:1–12. doi: 10.1038/ncomms4903, PMID: 24875164 PMC4075468

[46] Araki R Uda M Hoki Y Sunayama M Nakamura M Ando S . Negligible immunogenicity of terminally differentiated cells derived from induced pluripotent or embryonic stem cells. Nature. (2013) 494:100–4. doi: 10.1038/nature11807, PMID: 23302801

[47] Guha P Morgan JW Mostoslavsky G Rodrigues NP Boyd AS . Lack of immune response to differentiated cells derived from syngeneic induced pluripotent stem cells. Cell Stem Cell. (2013) 12:407–12. doi: 10.1016/j.stem.2013.01.006, PMID: 23352605

[48] Veevers J Farah EN Corselli M Witty AD Palomares K Vidal JG . Cell-surface marker signature for enrichment of ventricular cardiomyocytes derived from human embryonic stem cells. Stem Cell Rep. (2018) 11:828. doi: 10.1016/j.stemcr.2018.07.007, PMID: 30122443 PMC6135222

[49] Cyganek L Tiburcy M Sekeres K Gerstenberg K Bohnenberger H Lenz C . Deep phenotyping of human induced pluripotent stem cell–derived atrial and ventricular cardiomyocytes. JCI Insight. (2018) 3:e99941. doi: 10.1172/jci.insight.99941, PMID: 29925689 PMC6124434

[50] Scharler C Poupardin R Ebner-Peking P Wolf M Schreck C Brachtl G . Extra-hematopoietic immunomodulatory role of the guanine-exchange factor DOCK2. Commun Biol. (2022) 5:1–10. doi: 10.1038/s42003-022-04078-1, PMID: 36380073 PMC9666545

[51] Terheyden-Keighley D Hühne M Berger T Hiller B Martins S Gamerschlag A . GMP-compliant iPS cell lines show widespread plasticity in a new set of differentiation workflows for cell replacement and cancer immunotherapy. Stem Cells Transl Med. (2024) 13:898–911. doi: 10.1093/stcltm/szae047, PMID: 39042522 PMC11386223

[52] Dvorak P Bednar D Vanacek P Balek L Eiselleova L Stepankova V . Computer-assisted engineering of hyperstable fibroblast growth factor 2. Biotechnol Bioeng. (2018) 115:850–62. doi: 10.1002/bit.26531, PMID: 29278409

[53] Kuo HH Gao X DeKeyser JM Fetterman KA Pinheiro EA Weddle CJ . Negligible-cost and weekend-free chemically defined human iPSC culture. Stem Cell Rep. (2020) 14:256–70. doi: 10.1016/j.stemcr.2019.12.007, PMID: 31928950 PMC7013200

[54] Halloin C Schwanke K Löbel W Franke A Szepes M Biswanath S . Continuous WNT control enables advanced hPSC cardiac processing and prognostic surface marker identification in chemically defined suspension culture. Stem Cell Rep. (2019) 13:366–79. doi: 10.1016/j.stemcr.2019.06.004, PMID: 31353227 PMC6700605

[55] Kriedemann N Triebert W Teske J Mertens M Franke A Ullmann K . Standardized production of hPSC-derived cardiomyocyte aggregates in stirred spinner flasks. Nat Protoc. (2024) 19:1911–39. doi: 10.1038/s41596-024-00976-2, PMID: 38548938

[56] Burridge PW Matsa E Shukla P Lin ZC Churko JM Ebert AD . Chemically defined and small molecule-based generation of human cardiomyocytes. Nat Methods. (2014) 11:855. doi: 10.1038/nmeth.2999, PMID: 24930130 PMC4169698

[57] Frenzel LP Abdullah Z Kriegeskorte AK Dieterich R Lange N Busch DH . Role of natural-killer group 2 member D ligands and intercellular adhesion molecule 1 in natural killer cell-mediated lysis of murine embryonic stem cells and embryonic stem cell-derived cardiomyocytes. Stem Cells. (2009) 27:307–16. doi: 10.1634/stemcells.2008-0528, PMID: 18988711

[58] Nakamura Y Miyagawa S Yoshida S Sasawatari S Toyofuku T Toda K . Natural killer cells impede the engraftment of cardiomyocytes derived from induced pluripotent stem cells in syngeneic mouse model. Sci Rep. (2019) 9:10840–. doi: 10.1038/s41598-019-47134-3, PMID: 31346220 PMC6658523

[59] Parent AV Faleo G Chavez J Saxton M Berrios DI Kerper NR . Selective deletion of human leukocyte antigens protects stem cell-derived islets from immune rejection. Cell Rep. (2021) 36 (7):109538. doi: 10.1016/j.celrep.2021.109538, PMID: 34407395 PMC12416289

[60] Sung TC Jiang YP Hsu JY Ling QD Chen H Kumar SS . Transient characteristics of universal cells on human-induced pluripotent stem cells and their differentiated cells derived from foetal stem cells with mixed donor sources. Cell Prolif. (2021) 54:e12995. doi: 10.1111/cpr.12995, PMID: 33522648 PMC7941237

[61] Rossbach B Hariharan K Mah N Oh SJ Volk HD Reinke P . Human iPSC-Derived Renal Cells Change Their Immunogenic Properties during Maturation: Implications for Regenerative Therapies. Cells. (2022) 11:1328. doi: 10.3390/cells11081328, PMID: 35456007 PMC9032821

[62] Chimienti R Baccega T Torchio S Manenti F Pellegrini S Cospito A . Engineering of immune checkpoints B7-H3 and CD155 enhances immune compatibility of MHC-I–/– iPSCs for β cell replacement. Cell Rep. (2022) 40 (13):111423. doi: 10.1016/j.celrep.2022.111423, PMID: 36170817 PMC9532846

[63] Bogomiakova ME Sekretova EK Anufrieva KS Khabarova PO Kazakova AN Bobrovsky PA . iPSC-derived cells lack immune tolerance to autologous NK-cells due to imbalance in ligands for activating and inhibitory NK-cell receptors. Stem Cell Res Ther. (2023) 14:77–. doi: 10.1186/s13287-023-03308-5, PMID: 37038186 PMC10088155

[64] Boegel S Löwer M Bukur T Sorn P Castle JC Sahin U . HLA and proteasome expression body map. BMC Med Genomics. (2018) 11:36. doi: 10.1186/s12920-018-0354-x, PMID: 29587858 PMC5872580

[65] Ugolini F Szumera-Ciećkiewicz A Baroni G Nesi G Mandalà M Ferrone S . Differential HLA class I subunit (A, B, C heavy chain and β2-microglobulin) expression levels in normal tissues. Virchows Archiv. (2023) 482:359–68. doi: 10.1007/s00428-022-03459-5, PMID: 36437414 PMC9931818

[66] Didié M Galla S Muppala V Dressel R Zimmermann WH . immunological Properties of Murine Parthenogenetic stem cell-Derived cardiomyocytes and engineered heart Muscle. Front Immunol. (2017) 8:270621. doi: 10.3389/fimmu.2017.00955, PMID: 28855904 PMC5557729

[67] Yamamoto F Suzuki S Mizutani A Shigenari A Ito S Kametani Y . Capturing differential allele-level expression and genotypes of all classical HLA loci and haplotypes by a new capture RNA-seq method. Front Immunol. (2020) 11:941. doi: 10.3389/fimmu.2020.00941, PMID: 32547543 PMC7272581

[68] Carey BS Poulton KV Poles A . Factors affecting HLA expression: A review. Int J Immunogenet. (2019) 46:307–20. doi: 10.1111/iji.12443, PMID: 31183978

[69] Johansson T Partanen J Saavalainen P . HLA allele-specific expression: Methods, disease associations, and relevance in hematopoietic stem cell transplantation. Front Immunol. (2022) 13. doi: 10.3389/fimmu.2022.1007425, PMID: 36248878 PMC9554311

[70] Johansson T Yohannes DA Koskela S Partanen J Saavalainen P . HLA RNAseq reveals high allele-specific variability in mRNA expression. bioRxiv. (2018) 413534. doi: 10.1101/413534 PMC7949471 33717155

[71] Kawamura T Miyagawa S Fukushima S Maeda A Kashiyama N Kawamura A . Cardiomyocytes derived from MHC-homozygous induced pluripotent stem cells exhibit reduced allogeneic immunogenicity in MHC-matched non-human primates. Stem Cell Rep. (2016) 6:312–20. doi: 10.1016/j.stemcr.2016.01.012, PMID: 26905198 PMC4788782

[72] Wei L Lu J Feng L Long D Shan J Li S . HIF-1alpha accumulation upregulates MICA and MICB expression on human cardiomyocytes and enhances NK cell cytotoxicity during hypoxia-reoxygenation. Life Sci. (2010) 87:111–9. doi: 10.1016/j.lfs.2010.05.012, PMID: 20566410

[73] Lee JH Protze SI Laksman Z Backx PH Keller GM . Human pluripotent stem cell-derived atrial and ventricular cardiomyocytes develop from distinct mesoderm populations. Cell Stem Cell. (2017) 21:179–194.e4. doi: 10.1016/j.stem.2017.07.003, PMID: 28777944

[74] Mummery CL Zhang J Ng ES Elliott DA Elefanty AG Kamp TJ . Differentiation of human embryonic stem cells and induced pluripotent stem cells to cardiomyocytes: A methods overview. Circ Res. (2012) 111:344–58. doi: 10.1161/CIRCRESAHA.110.227512, PMID: 22821908 PMC3578601

[75] Churko JM Garg P Treutlein B Venkatasubramanian M Wu H Lee J . Defining human cardiac transcription factor hierarchies using integrated single-cell heterogeneity analysis. Nat Commun. (2018) 9:4906–. doi: 10.1038/s41467-018-07333-4, PMID: 30464173 PMC6249224

[76] Ebner-Peking P Krisch L Wolf M Hochmann S Hoog A Vári B . Self-assembly of differentiated progenitor cells facilitates spheroid human skin organoid formation and planar skin regeneration. Theranostics. (2021) 11:8430–47. doi: 10.7150/thno.59661, PMID: 34373751 PMC8344006

