# Supplementary material for: Deep immune-phenotyping of HLA-homozygous iPS-cardiomyocytes by spectral flow cytometry
Source: Front Immunol. 2026 Feb 4;17:1736994. doi: 10.3389/fimmu.2026.1736994 (PMC12913090; doi:10.3389/fimmu.2026.1736994)
Supplement: Supplementary file 1 [file DataSheet1.docx]

Supplementary Material

# Supplementary Figures and Tables

## Supplemetary Tables

**Supplementary Table S1: HLA haplotypes of iPSC lines used in this study**

|  |  | MHC I | | | MHC II | | |
| --- | --- | --- | --- | --- | --- | --- | --- |
|  |  | HLA-A | HLA-B | HLA-C | HLA-DRB1 | HLA-DQB1 | HLA-DPB1 |
| R26 | Allele 1 | 01:01:01 | 08:01:01 | 07:01:01 | 03:01:01G | 02:01:01G | 01:01:01 |
|  | Allele 2 | 01:01:01 | 08:01:01 | 07:01:01 | 03:01:01G | 02:01:01G | 04:01:01G |
| R26^DKO^ | Allele 1 | 01:01:01 | 08:01:01 | 07:01:01 | 03:01:01G | 02:01:01G | 01:01:01 |
|  | Allele 2 | 01:01:01 | 08:01:01 | 07:01:01 | 03:01:01G | 02:01:01G | 04:01:01G |
| MDCi246 | Allele 1 | 03:01:01 | 07:02:01 | 07:02:01 | 15:01:01G | 06:02:01G | 02:01:02G |
|  | Allele 2 | 03:01:01 | 07:02:01 | 07:02:01 | 15:01:01G | 06:02:01G | 02:01:02G |
| MDCi055 | Allele 1 | 01:01:01 | 08:01:01 | 07:01:01 | 01:01:01G | 03:02:01G | 04:01:01G |
|  | Allele 2 | 01:01:01 | 08:01:01 | 07:01:01 | 03:01:01G | 05:01:01G | 13:01:01 |
| PMU1 | Allele 1 | 03:01:01 | 15:09:01 | 03:04:01 | 04:03:01G | 03:02:01G | 03:01:01G |
|  | Allele 2 | 24:02:01 | 40:01:02 | 07:04:01 | 13:02:01 | 06:04:01G | 04:01:01G |

**Supplementary Table S2: Antibodies used for immunophenotyping of iPS-CM**

| **TARGET** | **FLUOROCHROME** | **CLONE** | **VENDOR** | **TUBE** |
| --- | --- | --- | --- | --- |
| Viability | Zombie NIR | n.a. | BioLegend | t1/2/3 |
| cTNT | Brilliant Violet 421 | 13-11 | BD | t1/2/3 |
| Tra-1-81 | Alex Fluor 488 | TRA-1-81 | BioLegend | t1 |
| SSEA-5 | RB545 | 8e11/SSEA-5 | BD | t1 |
| SSEA-4 | PE-Dazzle 594 | MC-813-70 | BioLegend | t1 |
| Tra-1-60-R | PE-Cy7 | TRA-1-60-R | BioLegend | t1 |
| CD31 | StarBright UV795 | WM59 | BioRad | t1 |
| CD44 | NovaFluor Red 700 | IM7 | eBioscience | t1 |
| CD142 (tissue factor) | BUV737 | HTF1 | BD | t1 |
| CD107a (LAMP-1) | APC-Fire 750 | H4A3 | BioLegend | t1 |
| CD45 | APC-Fire 810 | HI30 | BioLegend | t1 |
| CD34 | Pacific Blue | 581 | BioLegend | t1 |
| CD90 | Brilliant Violet 510 | 5E10 | BioLegend | t1 |
| CD140b (PDGFRβ) | Brilliant Violet 605 | 28D4 | BD | t1 |
| CD43 | Brilliant Violet 650 | 1G10 | BD | t1 |
| CD201 (EPCR) | Brilliant Violet 711 | RCR-252 | BD | t1 |
| Galectin-3 | Alexa Fluor 488 | B2C10 | BD | t2 |
| CD164 (MUC-24) | RB545 | N6B6 | BD | t2 |
| CD273 (PD-L2) | PE | MIH18 | BioLegend | t2 |
| HLA-F | PE-Dazzle 594 | 3D12 | BioLegend | t2 |
| CD80 (B7-1) | PerCP | MEM-233 | eBioscience | t2 |
| CD112 (Nectin-2) | PerCP-Cy5.5 | TX31 | BioLegend | t2 |
| MICA/B | PE-Cy7 | 6D4 | BioLegend | t2 |
| HLA-G | APC | 87G | eBioscience | t2 |
| CD86 (B7-2) | NovaFluor Yellow 690 | IT2.2 | eBioscience | t2 |
| CD47 | Alexa Fluor 700 | CC2C6 | BioLegend | t2 |
| CD155 (PVR) | BUV737 | SKII.4 | BD | t2 |
| CD30 (TNFRSF8) | APC-Fire 750 | BY88 | BioLegend | t2 |
| CD274 (PD-L1) | APC-Fire 810 | MIH3 | BioLegend | t2 |
| HLA-ABC | Pacific Blue | W6/32 | BioLegend | t2 |
| HLA-DR | eFluor 506 | LN3 | eBioscience | t2 |
| CD276 (B7-H3) | Brilliant Violet 605 | 7-517 | BD | t2 |
| HLA-E | Brilliant Violet 650 | 3D12 | BD | t2 |
| ICOSL | Brilliant Violet 711 | 2D3/B7-H2 | BD | t2 |
| CD137L (4-1BBL) | Brilliant Violet 786 | C65-485 | BD | t2 |
| CD55 (DAF) | Alexa Fluor 488 | MEM-118 | Invitrogen | t3 |
| CD66a (CEACAM1) | RB545 | B1.1/CD66 | BD | t3 |
| CD220 | PE | B6.220 | BioLegend | t3 |
| CD141 | PE-Dazzle 594 | M80 | BioLegend | t3 |
| CD59 (protectin) | PerCP | 029 | Invitrogen | t3 |
| CD36 (SCARB3) | PerCP-Cy5.5 | 5-271 | BioLegend | t3 |
| CD107b (LAMP-2) | PE-Cy7 | H4B4 | Novus Biologicals | t3 |
| CD13 | PE-Fire 810 | WM15 | BioLegend | t3 |
| Heme oxigenase 1 | APC | EPR1390Y | Abcam | t3 |
| CD46 | Alexa Fluor 700 | MEM-258 | Invitrogen | t3 |
| CD106 (VCAM-1) | BUV737 | 51-10C9 | BD | t3 |
| ROR1 | APC-Fire 750 | 2A2 | BioLegend | t3 |
| CD325 (N-cadherin) | BUV805 | 8C11 | BD | t3 |
| CD326 (EpCAM) | Pacific Blue | 9C4 | BioLegend | t3 |
| CD56 (NCAM) | SPARK YG 581 | QA17A16 | BioLegend | t3 |
| CD172a (SIRPα) | Brilliant Violet 605 | SE5A5 | BD | t3 |
| CD235a (GYPA) | Brilliant Violet 650 | HIR2 | BD | t3 |
| CD140a (PDGFRα) | Brilliant Violet 711 | 16A1 | BD | t3 |
| CD184 (CXCR4) | Brilliant Violet 785 | 12G5 | BioLegend | t3 |

## Supplementary Figures


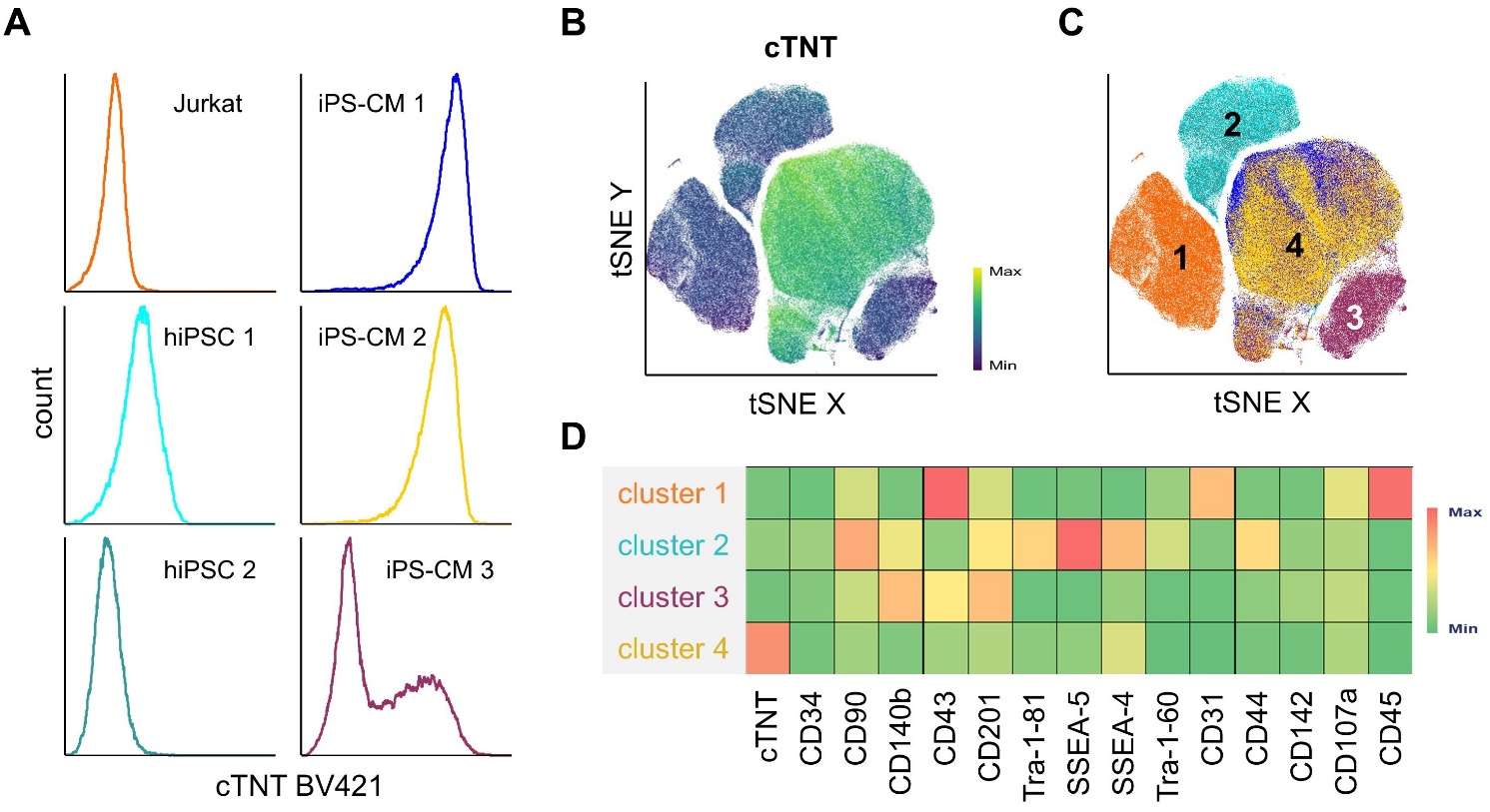


**Supplementary Figure S1. QC panel run on iPS-CM, hiPSC, and Jurkat cells. (A)** Histograms showing cTNT expression of Jurkat cells, hiPSC, and iPS-CM stained with the 16-marker quality control panel. **(B)** Clustering of samples from (A) by tSNE and colored according to cTNT expression. **(C)** Depiction of cluster 1-4 by sample identity. Samples are color-coded as seen in (A). **(D)** Marker expression profile of individual clusters shown in (C).


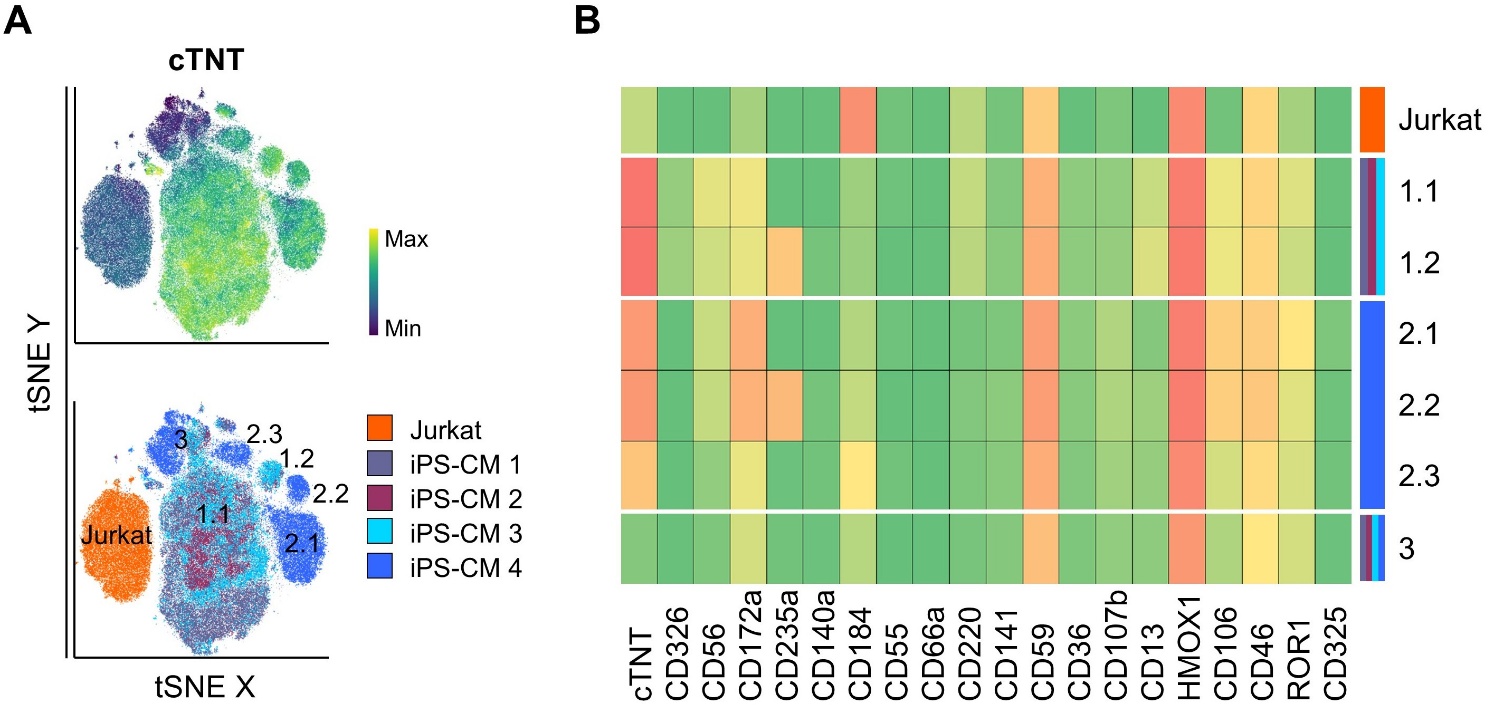


**Supplementary Figure S2. Extended immune phenotyping of iPS-CMs.** (**A**) Dimensionality reduction (tSNE) of Jurkat cells and four independent iPS-CM batches based on the extended 21-marker expression profile t3. Upper: Representation of cTNT expression by signal heat. Lower: Representation by sample identity. **(B)** Marker expression profile of individual clusters shown in (A).


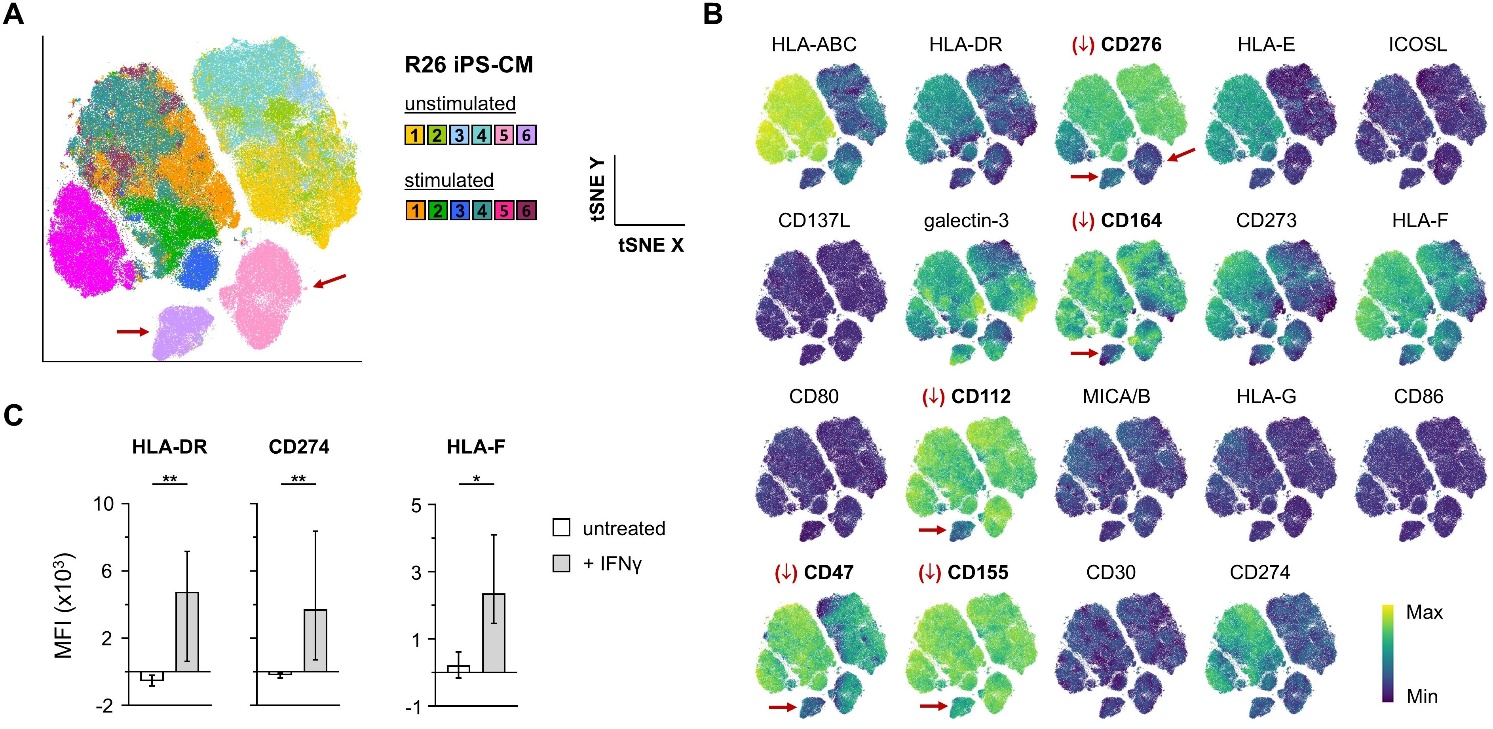


**Supplementary Figure S3: Outlier detection using the 21-color immune recognition tube t2.** (**A**) Dimensionality reduction (tSNE) of 6 independent differentiation runs using R26. Color-coding represents sample identities. **(B)** Clustering from (A) resolved by expression heat of the individual markers in panel p2. (**C**) Statistical analysis of selected markers in untreated and IFNγ-treated samples. Paired Student’s t-test, two-tailed; ** p ≤ 0.01, *** p ≤ 0.001
